# Quantitative Shape Irregularity and Density Heterogeneity Predict Hematoma Expansion in Patients With Intracerebral Hemorrhage

**DOI:** 10.1002/acn3.70141

**Published:** 2025-07-14

**Authors:** Zeqiang Ji, Yunyi Hao, Bin Gao, Xiaojing Zhang, Yani Zhang, Jiaokun Jia, Xue Xia, Yuhao Guo, Sijia Li, Jianwei Wu, Kaijiang Kang, Xingquan Zhao

**Affiliations:** ^1^ Department of Neurology Beijing Tiantan Hospital, Capital Medical University Beijing China; ^2^ China National Clinical Research Center for Neurological Diseases Beijing China; ^3^ Beijing Stroke Association Beijing China; ^4^ Department of Clinical Epidemiology and Clinical Trial Capital Medical University Beijing China; ^5^ Department of Internal Medicine MedStar Washington Hospital Center Washington DC USA; ^6^ Department of Epidemiology Beijing Neurosurgical Institute, Beijing Tiantan Hospital, Capital Medical University Beijing China; ^7^ Department of Neurosurgery Beijing Tiantan Hospital, Capital Medical University Beijing China

**Keywords:** hematoma auto‐segmenting, hematoma expansion, hematoma surface and density characteristics, intracerebral hemorrhage

## Abstract

**Purpose:**

This study aimed to explore the association between quantitative shape irregularity and density heterogeneity of hematomas and hematoma expansion (HE) for intracerebral hemorrhage (ICH) patients.

**Methods:**

This cohort study included patients arriving within 24 h of symptom onset between August 2021 and July 2022 as the derivation cohort and those between July 2023 and February 2024 as the external validation cohort. HE is defined as a hematoma increase of > 6 mL or > 33% from the baseline to the follow‐up CT scan between 24 and 48 h. The least absolute shrinkage and selection operator (LASSO) regression was applied to select the traditional image signs to fit the logistic regression as Model 1. Afterwards, the surface regularity index (SRI) and density coefficient of variation (DCV) of hematoma were added to form Model 2. Finally, we used the SRI and DCV to replace the selected traditional image signs as Model 3. The performance and clinical utilities were evaluated and compared in the external validation cohort.

**Result:**

The three models demonstrated good discrimination in both the derivation cohort and the validation cohort, with Model 2 and Model 3 showing significant improvements in area under the receiver operating characteristic curve (AUROC) and in clinical utility compared to Model 1 (Model 2 AUROC: 0.859 [95% CI: 0.802, 0.926] vs. Model 1 AUROC: 0.713 [95% CI: 0.625, 0.814], Delong test *p* < 0.001; Model 3 AUROC: 0.840 [95% CI: 0.776, 0.912] vs. Model 1 AUROC: 0.713 [95% CI: 0.625, 0.814], *p* = 0.006). The SRI and DCV can improve the prediction of HE based on traditional clinical indicators and imaging signs, also serving as possible alternatives to traditional imaging signs.

**Conclusions:**

The SRI and DCV can serve as effective substitutes for traditional imaging signs in predicting hematoma expansion.

## 1 Introduction

Intracerebral hemorrhage (ICH) is a severe subtype of stroke, constituting 10%–30% of all strokes globally, with a 30‐day mortality rate ranging from 30% to 40% [1, 2]. Many survivors experience disabilities, resulting in a substantial healthcare burden [3]. Hematoma expansion (HE) is a primary contributor to early neurological deterioration (END) and unfavorable functional outcomes in patients with ICH [4, 5]. The precise identification of high‐risk HE patients has long been a pivotal focus in the management of ICH [6]. Several radiological indicators, like the computed tomography angiography (CTA) spot sign and non‐contrast computed tomography (NCCT) signs, have been developed to stratify patients at high risk of HE with acceptable discriminations [7, 8]. However, the limited accessibility of CTA, coupled with the low detection rate of the CTA spot sign, has restricted its application in clinical practice [9]. Concurrently, these NCCT signs are qualitative, and the interpretation is mostly subjective, leading to suboptimal accuracy for predicting HE [10]. Therefore, it is of great clinical significance to develop a reliable predictive model for the early detection of high‐risk HE cases.

The 3D‐Slicer software (www.slicer.org, Harvard University, USA) has been previously utilized for hematoma modeling in studies of ICH, offering objective quantitative assessment of hematoma shape irregularity and density variability [11]. However, its application in clinical scenarios that necessitate rapid decision‐making, such as emergency care, has been constrained by the labor‐intensive manual segmenting processes employed. The recently developed MONAI label for artificial intelligence recognition can accomplish hematoma segmentation effectively within the circumstances of 3D‐Slicer software, which enables the rapid acquisition of objective indicators regarding hematoma characteristics to facilitate predictive analysis [12]. Although recent studies have demonstrated the efficacy of deep learning in hematoma segmentation and HE prediction, a universally accepted artificial intelligence (AI) method for predicting HE still remains absent due to the “black box” nature of AI predictive models and their suboptimal performance [13, 14]. Consequently, integrating AI‐driven hematoma segmentation methods with the interpretation of hematoma characteristics to develop prediction nomograms may represent a promising and valuable approach for HE management. Herein, we aimed to identify new HE predictors in patients with ICH and develop a novel predictive nomogram.

## 2 Methods

### 2.1 Patient Selection

This was a prospective, observational cohort study of ICH patients admitted to Beijing Tiantan Hospital. The derivation cohort comprised eligible ICH patients included between August 2021 and July 2022, while the external validation cohort comprised ICH patients enrolled from July 2023 to February 2024. The inclusion criteria were (1) age from 18 to 80 years; (2) baseline NCCT and CTA were completed within 24 h of onset; (3) follow‐up NCCT was completed from 24 to 48 h after symptom onset. The exclusion criteria were: (1) traumatic brain bleeding; (2) ICH related to arteriovenous malformation, aneurysm, or neoplastic lesion; (3) hemorrhagic transformation after ischemic stroke; (4) primary interventricular hemorrhage; (5) previous anticoagulant use; (6) critical medical records or follow‐up image missed. The study was performed by the guidelines of the Helsinki Declaration and approved by the Research Ethics Committee of Beijing Tiantan Hospital (KY2021‐025‐01). Written informed consent was obtained from all subjects or their legally authorized representatives.

### 2.2 Data Collection

The clinical data was recorded in the prospective cohort database, including demographic characteristics (sex and age), medical history (hypertension, diabetes mellitus, coronary heart disease, previous antiplatelet use), baseline neurological scores (GCS and NIHSS), radiological characteristics (hematoma location, volume, presence of intraventricular hemorrhage, and time of baseline CT). Blood samples were drawn from an antecubital vein within 30 min of arrival and before any treatment. Laboratory tests included hematologic tests (admission sodium, potassium, glucose level; leukocyte, neutrophil, lymphocyte and platelet count, brain natriuretic peptide (BNP), D‐dimer, etc.).

### 2.3 Definition and Measurement of Hematoma Shape and Density

Baseline and follow‐up NCCT (16‐slice CT scanner, Brilliance iCT, Philips Healthcare, Cleveland, OH) were performed using the following helical scan parameters: 120 kVp, 310 mAs, 1 mm thickness, image size: 512 × 512. The hematoma location and presence of interventricular hemorrhage were documented.

**FIGURE 1:**
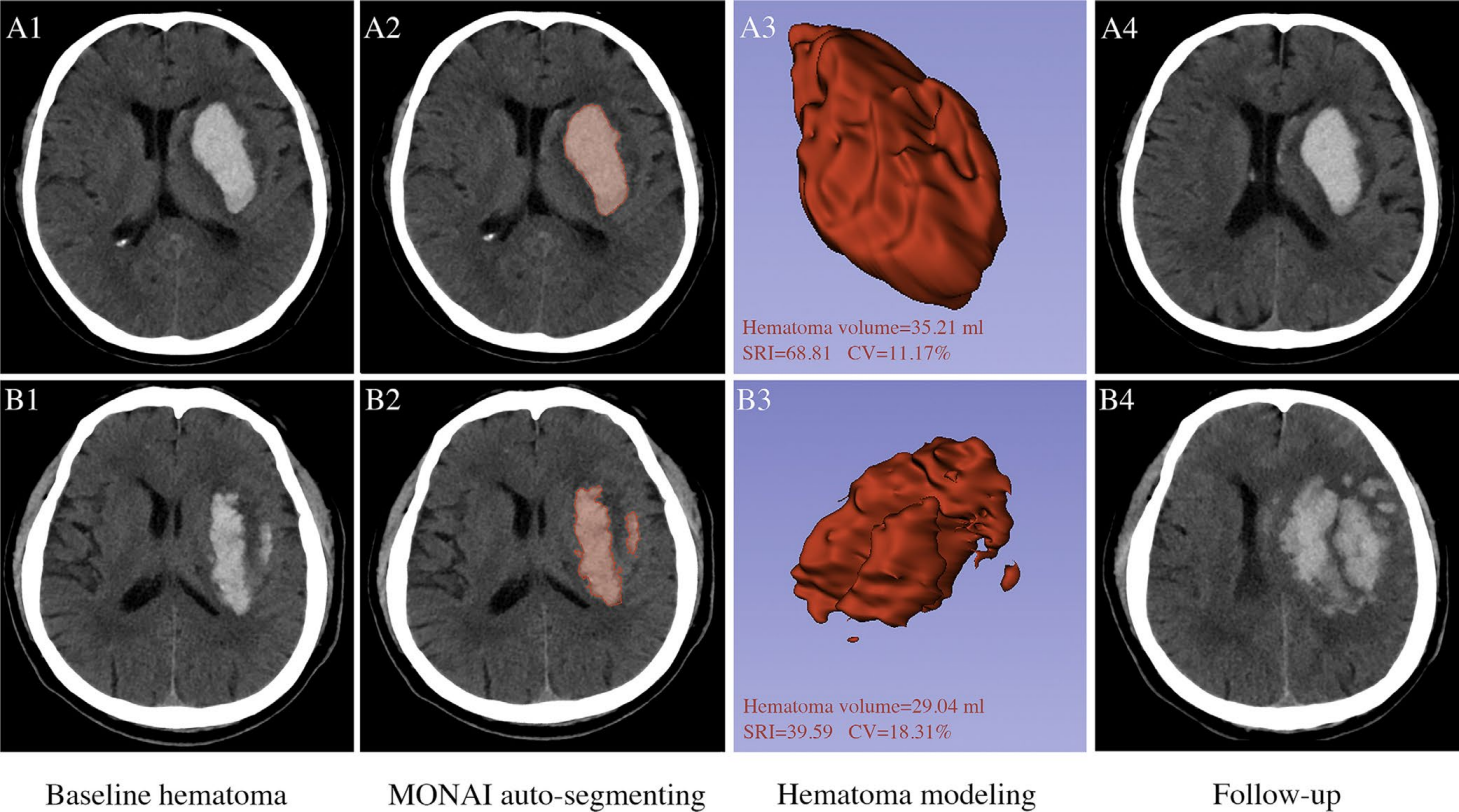
Segmenting and modeling of hematoma utilizing the MONAI label. A1–A4 are images taken from an ICH patient with a high SRI and low DCV of baseline hematoma who did not experience HE. B1–B4 are images taken from an ICH patient with low SRI and high DCV who had HE in follow‐up CT. A1–A3 and B1–B3 show hematoma segmentation and modeling performed using the MONAI label integrating in the 3D‐Slicer software based on baseline CT. The results of the modeled hematoma measurements and calculations (hematoma volume, SRI, and DCV) are presented in A3 and B3. CT, computed tomography; DCV, density coefficient of variation; ICH, intracerebral hemorrhage; SRI, surface regularity index.

The hematoma was segmented and reconstructed from the DICOM data of the baseline CT using the 3D‐Slicer software (version 5.6.2, www.slicer.org, Harvard University, USA) (Figure 1). The hematoma was automatically identified via the MONAI label (the “intracerebral hemorrhage” model, specifically). Simultaneously, the automatically generated segmentation can be manually revised as necessary to enhance the precision of labeling [12]. The automatic calculation, along with manual modification and confirmation, can generally be completed within an average of 3 min.

Finally, the Models module was utilized to reconstruct the three‐dimensional data by adding up all the pixels from each slice, and the values of hematoma volume and surface area were directly obtained from the 3D‐Slicer without smoothing processing. The surface regularity index (SRI) was calculated from using the following formula [15]:

$$\mathrm{SRI}=6\sqrt{\pi }\left(\frac{\text{hematoma volume}}{\sqrt{{\left(\text{hematoma surface area}\right)}^{3}}}\right)\times 100$$

We also used the “Statistics” modules to automatically calculate the mean and standard deviation (SD) of the CT values of each pixel. The density coefficient of variation (DCV) was calculated as follows to represent the hematoma density heterogeneity of the hematoma. The entire process takes an average of about 5 min.

$$\mathrm{DCV}=\left(\frac{\text{Standard Deviation}}{\text{Mean}}\right)\times 100\%$$

### 2.4 Radiological Signs Interpretation

The computed tomography angiography (CTA) spot signs [7] and various NCCT signs, including the hypodensities, blend sign, black hole sign, island sign, satellite sign, heterogeneous, and irregular shape, were interpreted based on their definitions [8]. All radiological signs were identified by two trained neurologists who were blinded to the outcomes and were supervised by a veteran radiologist. The consistency test was carried out, and the formal interpretation started when the intra‐class correlation coefficient (ICC) reached above 0.8.

## 3 Outcomes

HE was defined as an increase of > 6 mL absolute volume or > 33% relative hematoma volume from the baseline to the follow‐up CT scan between 24 and 48 h [16].

## 4 Statistical Analysis

Continuous variables were expressed as median (interquartile range, IQR) or mean (SD) and examined using the Mann–Whitney *U*‐test or Student's *t*‐test. Categorical variables are presented as counts (percentages) and were compared using the *χ* ^2^ test or Fisher's exact test. Differences with *p* < 0.05 were considered statistically significant for two‐tailed tests.

In the derivation cohort, to compare the predictive performance of traditional radiological signs and quantitative imaging biomarkers (DCV and SRI) for HE prediction, we conducted variable selection for candidate variables and developed two separate predictive models. The candidate variable set for Model 1 encompasses clinical variables and radiological signs, including CTA spot sign, hypodensities, blend sign, black hole sign, island sign, CTA spot sign, satellite sign, irregular, and heterogeneous. Variable selection was conducted using a Least Absolute Shrinkage and Selection Operator (LASSO) penalized regression model, and 5‐fold cross‐validation was employed to select the tuning parameters in the model [17]. Adhering to the 10 EPV principle and considering the sample size limitations of our derivation cohort, we selected the top seven variables with the largest absolute coefficients in the LASSO regression under the optimal tuning parameters. To ensure coefficient comparability, we applied one‐hot encoding to categorical variables and standardized numerical variables prior to regression analysis. Subsequently, we added DCV and SRI indicators to Model 1 as Model 2 to evaluate whether incorporating quantitative imaging biomarkers with traditional imaging signs would improve model performance. Finally, we replaced the traditional imaging signs in Model 1 with SRI and DCV as Model 3 to determine if quantitative imaging signs could replace traditional imaging signs to simplify the model. We fitted three models using logistic regression and presented them using nomograms. We conducted internal validation using a 5‐fold cross‐validation approach in the derivation cohort and then performed external validation in the validation cohort.

Model performance was evaluated in terms of discrimination, calibration, and clinical utility. Discrimination was measured using the area under the receiver operating characteristic curve (AUROC). Calibration was assessed visually by plotting the predicted versus observed risk of HE. The net benefit of models in clinical application processes across various threshold probabilities was presented using decision curve analysis (DCA). We employ the Delong test to compare the AUROC among three models and utilize the continuous Net Reclassification Index (NRI) and Integrated Discrimination Improvement (IDI) to assess the improvement in model performance. The 95% confidence intervals were estimated using a bootstrap method with 5000 resamples. All statistical analyses were performed using R (version 4.4.1).

## 5 Result

### 5.1 Participants Baseline Characteristics

**FIGURE 2:**
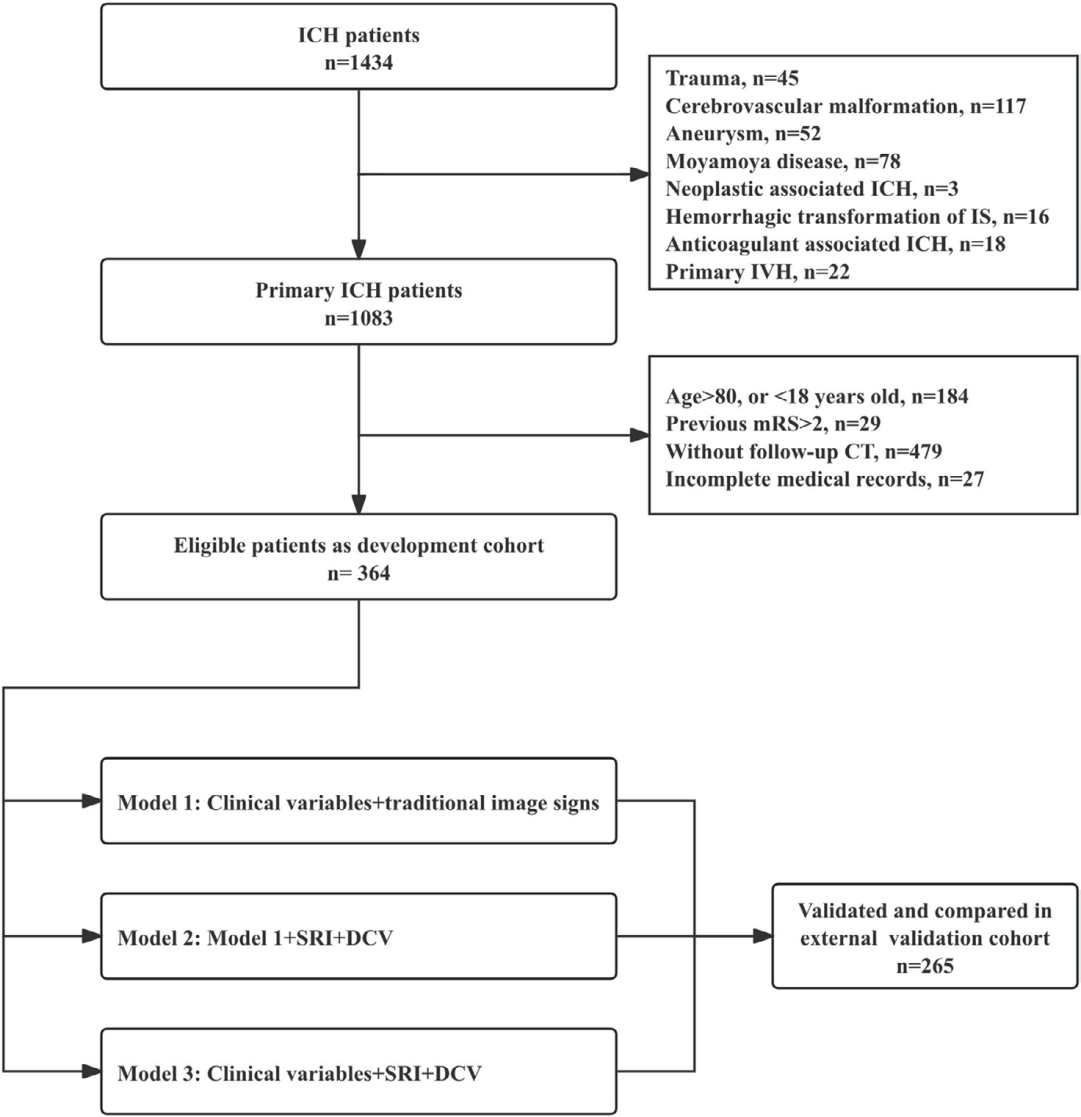
Flowchart of participants selection and graphic illustrations of this study. CT, computed tomography; DCV, density coefficient of variation; ICH, intracerebral hemorrhage; IVH, interventricular hemorrhage; mRS, modified Rankin Scale, SRI, surface regularity index.

A total of 364 patients diagnosed with ICH were included in the derivation cohort, of whom 70 (19.2%) experienced HE. Among them, 265 (72.8%) were male, and the median age was 56.0 years (IQR: 19.0). A flowchart of the patient selection process is shown in Figure 2. The median baseline GCS and NIHSS scores were 15 (IQR: 3) and 10 (IQR: 12), respectively. The median time from ICH onset to emergency was 5.6 h (IQR: 3.2). The median baseline hematoma volume was 23.8 mL (IQR: 22.8), with 85 patients found to be complicated with IVH. Deep hematomas (including the basal ganglia and thalamus) accounted for 75.9% of all hemorrhages, whereas lobar, cerebellar, and brainstem hematomas accounted for 19.2%, 3.9%, and 1.9%, respectively. The median SRI and DCV of baseline hematoma were 69.8 (IQR: 53.7) and 12.1% (IQR: 2.4), respectively. All the baseline characteristics are presented in Table S1.

### 5.2 Factors Associated With Hematoma Expansion

In terms of traditional radiological signs, the patients with HE exhibited a higher prevalence of CTA spot sign (20.0% vs. 5.1%, *p* < 0.001), hypodensities (62.9% vs. 42.5%, *p* = 0.002), satellite sign (32.9% vs. 21.1%, *p* = 0.036), heterogeneous (25.7% vs. 11.2%, *p* = 0.002), and irregular shape (32.9% vs. 19.4%, *p* = 0.014).

In the quantitative analysis of hematomas, patients with HE exhibited a more irregular morphology, as indicated by the SRI (56.9 vs. 75.9, *p* < 0.001), and demonstrated increased density heterogeneity, as reflected by DCV (13.5% vs. 11.9%, *p* < 0.001). No significant differences were observed in hematoma volume, incidence of IVH, or hematoma location between the two groups (Table S1).

### 5.3 Feature Selection and Model Development

**FIGURE 3:**
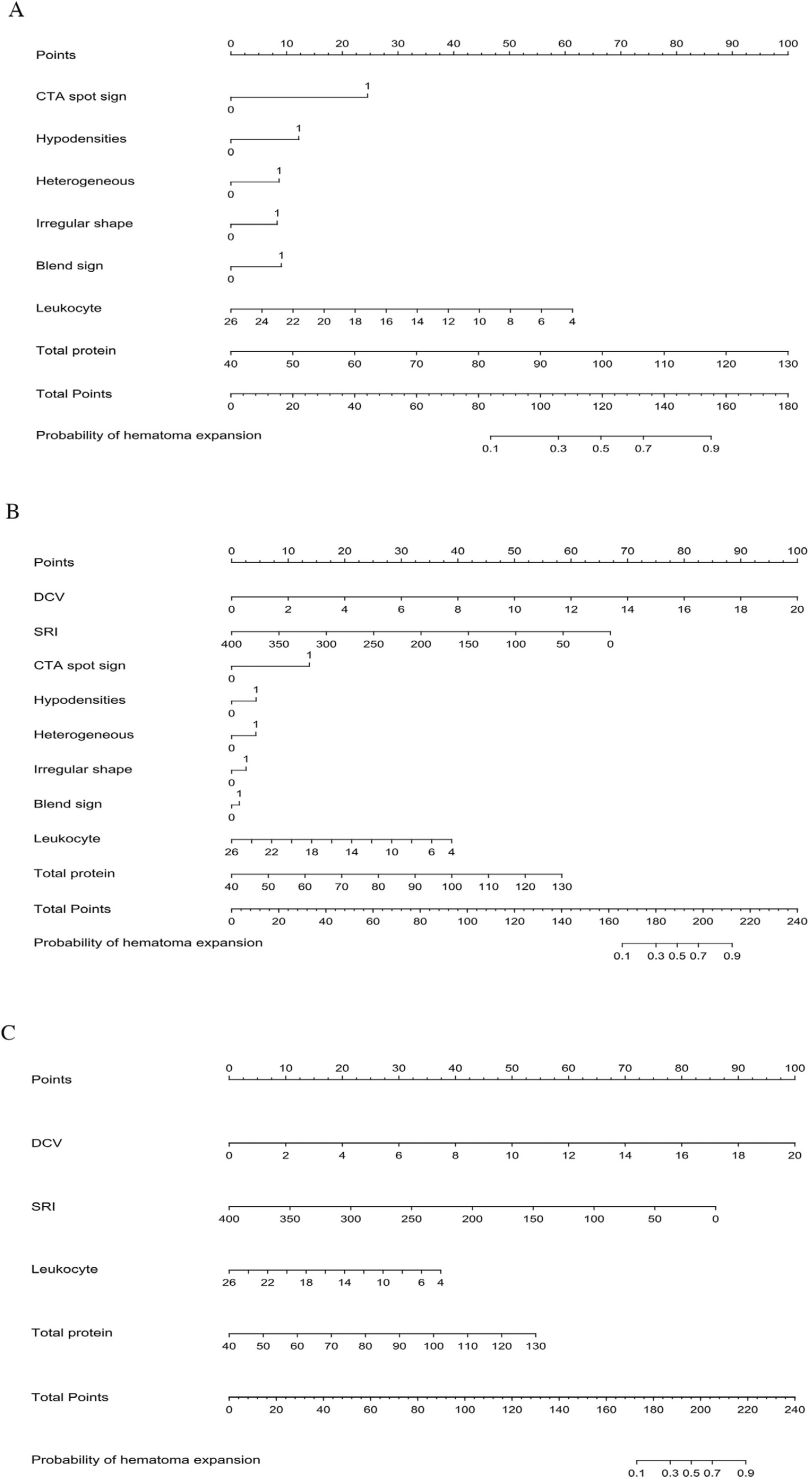
The predictive nomograms for hematoma expansion in patients with intracerebral hemorrhage. Model 1 (A), Model 2 (B) Model 3 (C). CTA, computed tomography angiography; DCV, density coefficient of variation; SRI, surface regularity index.

In the derivation cohort, we employed LASSO regression to select the most influential variables for Model 1, utilizing the minimum error criterion to determine the optimal lambda (*λ*) values. For Model 1, the optimal *λ* was identified as 0.0176 (Figure 1A). The selection of variables was conducted based on the absolute values of the coefficients, arranged in descending order under the optimal λ condition (Figure 1B). In the end, Model 1 contained CTA spot sign, hypodensities, blend sign, irregular shape, heterogeneous shape, leukocyte count, and total protein (Figure S1C). We incorporated SRI and DCV into Model 1 to create Model 2, and then we substituted traditional image indicators with SRI and DCV in Model 1 to form Model 3. The coefficients obtained from the Logistic regression models (Table S2) were utilized to generate nomograms for both models (Figure 3A for Model 1, Figure 3B for Model 2, Figure 3C for Model 3).

### 5.4 Model Performance and Validation

The performance of the three models in terms of discriminability and the comparative results of the models are presented in Figure 1A,B and Table 1. In the derivation cohort, all three models demonstrated good discrimination. However, during external validation, the AUROC of model 1 decreased, while no significant changes were observed in the other two models, indicating that the predictive models using SRI and DCV have good robustness. Notably, during external validation, model 2 showed a superior AUROC compared to Model 1 (Model 2 AUROC: 0.859 [95% CI: 0.802, 0.926] vs. Model 1 AUROC: 0.713 [95% CI: 0.625, 0.814], Delong test *p* < 0.001), suggesting that using SRI and DCV optimizes the predictive performance of the model based on traditional clinical indicators and imaging signs. Additionally, model 3 also showed significant improvement in discrimination compared to Model 1 (Model 3 AUROC: 0.840 [95% CI: 0.776, 0.912] vs. Model 1 AUROC: 0.713 [95% CI: 0.625, 0.814], Delong test *p* = 0.006), and there was no significant decrease in AUROC compared to Model 2 (Model 3 AUROC: 0.840 [95% CI: 0.776, 0.912] vs. Model 2 AUROC: 0.859 [95% CI: 0.802, 0.926], Delong test *p* = 0.006), indicating that SRI and DCV can replace traditional imaging signs for predicting hematoma expansion. The results of continuous NRI and IDI (Table 1) also show significant improvements in model 2 and model 3 compared to model 1.

**TABLE 1:** Predictive performance of models in derivation and validation cohorts.

Comparision	Delong test	NRI	IDI
Derivation cohort			
Model 2 versus Model 1	0.002	0.755 (0.561, 1.188)	0.157 (0.079, 0.254)
Model 3 versus Model 1	0.057	0.631 (0.448, 1.298)	0.144 (0.067, 0.299)
Model 3 versus Model 2	0.135	−0.165 (−0.408, 0.392)	−0.013 (−0.036, 0.078)
Validation cohort			
Model 2 versus Model 1	*p* < 0.001	1.018 (0.619, 1.355)	0.174 (0.020, 0.277)
Model 3 versus Model 1	0.006	0.585 (0.120, 1.455)	0.107 (−0.047, 0.299)
Model 3 versus Model 2	0.176	−0.706 (−1.142, −0.271)	−0.067 (−0.110, 0.061)

Abbreviations: IDI, integrated discrimination index; NRI, net reclassification index.

The calibration curve (Figure S1D,E) indicates that all three models exhibit good calibration in both internal and external validation, suggesting a good alignment between the predicted probabilities of the models and the actual occurrence probabilities of HE.

**FIGURE 4:**
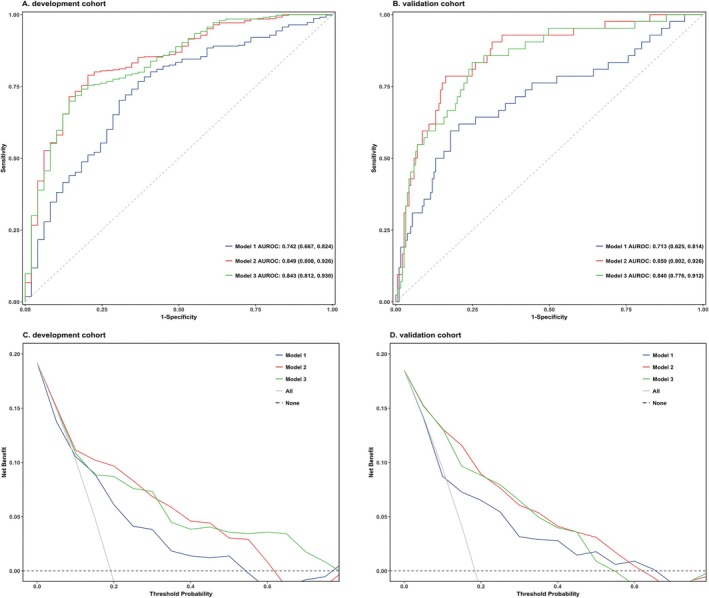
Predictive performance of models. ROC curves and comparison of AUROC between two models for predicting HE in development cohort (A) and validation cohort (B). DCA of 2 models was conducted to evaluate the predictive performance of HE in development cohort (C) and validation cohort (D). ROC, receiver operating characteristic curve; AUROC, areas under the curve of receiver operating characteristic curve; HE, hematoma expansion; DCA, decision curve analyses.

The decision curve analysis in both the derivation and validation cohort (Figure 4C,D) indicates that within the threshold probability range of 0.15–0.5, all three models exhibit a superior clinical net benefit compared to both the “All treat” and “None treat” strategies. Furthermore, within this range, Model 2 and Model 3 demonstrate a higher net benefit than Model 1, highlighting the advantage of employing SRI and DCV in constructing predictive models for clinical utility.

## 6 Discussion

To the best of our knowledge, this is the first study to quantitatively and objectively explore the association between shape irregularity, density heterogeneity of hematoma, and HE, utilizing an AI‐assisted interactive labeling. In this study, we developed and validated a nomogram with hematoma SRI and DCV to predict HE, achieving excellent predictive performance and a significant improvement compared to conventional radiological signs.

Hematoma expansion has been demonstrated to correlate with poor functional outcomes and various complications, including secondary perihematomal edema expansion, herniation, stroke‐associated pneumonia, and thrombotic events, which increase the risk of mortality and disability [5, 18, 19]. Concurrently, early initiation of intensive management, like antihypertensive therapy, has been indicated to have a positive impact on the prognosis of ICH patients [20]. Therefore, it is crucial to develop an accessible predicting model and timely identify patients at high risk of HE for the precise management of patients with ICH, which may lead to improved clinical outcomes [20].

Currently, the predictive methodologies for HE predominantly encompass variables such as clinical laboratory assessments [21, 22], imaging biomarkers (notably the well‐established CTA spot sign and NCCT signs) [23, 24], and predicting models based on the radiomic and deep learning algorithm [14, 25, 26]. Among these approaches, radiologic biomarkers are most utilized to predict HE and elucidate the underlying pathophysiological mechanisms, owing to their simplicity, rapidity, and intuitive depiction of hematomas [8]. For instance, the CTA spot sign is regarded as an indicator of contrast leakage within the hematoma, suggesting active bleeding [7], and the NCCT signs are typically characterized by irregular shapes and density heterogeneity within the hematoma, reflecting the dynamic process of hemorrhagic events [24].

Although the exact mechanism of HE remains unclear, the Fisher's domino model first demonstrated the associations between the irregular shape and HE. According to Fisher's theory, HE is caused by the shearing stress of blood vessels around the site of the primary hemorrhage, which contributes to the secondary hemorrhage [27]. Furthermore, active hematomas can modify their growth trajectories across various axial planes and are more susceptible to developing along diverse pathways with irregular margins. While the density heterogeneity generally indicates multiple origins of bleeding within a single hematoma at different times and spaces [28]. Consequently, imaging indicators derived from these theories, like spot sign and NCCT signs, have shown acceptable predictive abilities, but certain limitations persist. For instance, the CTA spot sign detection rate decreases as time‐to‐CTA increases (in our study, the detection rate of spot sign was only 20% in HE group) [9]. While the NCCT sign is relatively more accessible, the predictive performance and sensitivity remain suboptimal. This may be attributed to the subjective interpretation of the NCCT sign and a certain degree of conceptual overlap, which hinders its ability to serve as a standardized and objective metric for assessing hematoma morphology [8]. Therefore, developing standardized and objective metrics to delineate the heterogeneity in hematoma shape and density is of considerable importance.

The SRI could reflect morphological characteristics of the hematoma while DCV assesses density variability, offering a more objective and quantitative alternative compared to traditional NCCT signs [15]. Concurrently, the MONAI label utilized for hematoma segmentation enables us to delineate the hematoma boundaries objectively and effectively, which also enhances the reliability of those two indices. In our study, SRI and DCV could independently predict HE, improving predictive performance of the nomogram compared with the traditional model.

Several models had been developed utilizing deep learning algorithms to identify and delineate the hematoma and predict the possibility of HE [14, 26]. However, the current AI prediction tools have been limited in clinical practice due to the following issues: (1) the suboptimal predictive performance; (2) the “black box” nature; (3) lack of a unified and widely recognized method; (4) poor accessibility since most AI tools are driven by economic interests. Notably, the MONAI label integrated within 3D‐Slicer, a widely used and open‐source software in hematoma processing, exhibits robust capability for recognizing hematoma edges promptly (within 1 min for a single hematoma as in our study). The MONAI label enables rapid identification of the shape and density characteristics of hematoma in emergency or other clinical scenarios, which may serve as a useful tool for predicting HE [12]. In this study, the predictive model established based on SRI and DCV had demonstrated a relatively optimal predictive performance compared to the existing models [14, 25, 29, 30, 31]. Given the challenges associated with developing deep learning models and their low accessibility, it may prove beneficial to leverage deep learning techniques to identify parameters such as hematoma SRI and DCV for more accurate predictions of HE. An accurate prediction method for HE in the acute phase of intracerebral hemorrhage can help precisely identify high‐risk patients, facilitate targeted interventions including intensive blood pressure control [32], and ultimately improve the prognosis for these patients [33]. We believe that patients with low SRI and high DCV of the hematoma are at high risk for hematoma expansion. These patients should follow the intensive blood pressure control, hemostatic therapy, and more aggressive surgical options mentioned in the Code ICH consensus to improve patient outcomes [34].

Notably, we included ICH patients within 24 h of symptom onset, while the primary application scenario for spot signs and NCCT signs was limited to 6 h from onset to image [8, 10, 35]. In clinical practice, patients between 6 and 24 h of symptom onset still have a probability of experiencing HE (in our study, this probability was found to be 17.1%), and our models have extended the time window of imaging for predicting HE. Also, we performed subgroup analyses of the model's performance based on whether the time from intracerebral hemorrhage onset to CT was less than 6 h. The results showed that the model's predictive efficacy did not differ statistically across different time intervals (Delong test, in Figure S2A–C).

Some limitations of this study should be considered when interpreting the results. Firstly, our findings were derived from a single‐center analysis with specific inclusion and exclusion criteria. Therefore, a potential selection bias may be inevitable. Further multicenter, prospective studies are warranted for further validation. Secondly, SRI and DCV must be acquired via manual image processing, which may limit their clinical application. Automatic analysis software that calculates and processes these parameters will be developed in future studies.

## 7 Conclusion

The predictive nomograms based on the quantitative shape irregularity and density heterogeneity of baseline hematoma for HE exhibited stronger discrimination, accuracy, and potential clinical applicability in the future, compared to traditional radiological signs.

## Author Contributions

All authors made a significant contribution to the work reported, whether that is in the conception, study design, execution, acquisition of data, analysis and interpretation, or in all these areas; took part in drafting, revising, or critically reviewing the article; gave final approval of the version to be published; have agreed on the journal to which the article has been submitted; and agree to be accountable for all aspects of the work. All authors have read and approved the final submitted manuscript.

## Conflicts of Interest

The authors declare no conflicts of interest.

## Supporting information


Data S1.

## Data Availability

The data supporting the findings of this study are available from the corresponding author and the institution upon reasonable request.
