# Supplementary material for: Quantitative Shape Irregularity and Density Heterogeneity Predict Hematoma Expansion in Patients With Intracerebral Hemorrhage
Source: Ann Clin Transl Neurol. 2025 Jul 14;12(10):2068–76. doi: 10.1002/acn3.70141 (PMC12516230; doi:10.1002/acn3.70141)
Supplement: Supplementary file 1 — Data S1. [file ACN3-12-2068-s001.docx]

**Table S1. Baseline clinical and radiological characteristics of patients with and without hematoma expansion in derivation dataset.**

| **Characteristic** | **Total**  **(n=364)** | **non-HE (n=294)** | **HE  (n=70)** | **p** |
| --- | --- | --- | --- | --- |
| Age, median (IQR), y | 56.0 (19) | 56.0 (19.2) | 55.0 (22.0) | 0.954 |
| Sex |  |  |  | 0.364 |
| Male | 265 (72.8) | 211 (71.7) | 54 (77.1) |  |
| Female |  | 83 (28.3) | 16 (22.9) |  |
| Hypertension | 263 (72.3) | 212 (72.1) | 51 (72.9) | 0.900 |
| Diabetes mellitus | 59 (16.2) | 43 (14.6) | 16 (22.9) | 0.093 |
| CHD | 21 (5.8) | 15 (5.1) | 6 (8.6) | 0.263 |
| Ischemic stroke | 30 (8.2) | 30 (8.2) | 30 (8.2) | 0.392 |
| Previous antiplatelet therapy | 34 (9.3) | 28 (9.5) | 6 (8.6) | 0.806 |
| Baseline GCS, median (IQR) | 15 (3) | 15 (3) | 15 (3) | 0.986 |
| Baseline NIHSS, median (IQR) | 10 (12) | 9 (11) | 11 (12) | 0.149 |
| Baseline SBP, median (IQR), mmHg | 170 (30) | 170 (29) | 167 (34) | 0.495 |
| Onset to baseline CT, median (IQR), hours | 5.6 (3.2) | 6.0 (4) | 5.3 (4.5) | 0.023 |
| Time from onset to baseline CT |  |  |  | 0.508 |
| ≤6 hours | 224 (61.5) | 178 (60.5) | 46 (65.7) |  |
| >6 hours | 140 (38.5) | 116 (39.5) | 24 (34.3) |  |
| Hematoma volume, median (IQR), mL | 23.8 (22.8) | 22.8 (23.4) | 28.0 (22.0) | 0.217 |
| Hematoma surface area, median (IQR), cm2 | 52.8 (53.3) | 49.6 (54.8) | 67.1 (39.8) | 0.003 |
| Hematoma mean density, median (IQR), HU | 60.1 (4.6) | 60.3 (4.4) | 59.7 (1.6) | 0.037 |
| Hematoma density SD, median (IQR), HU | 7.3 (1.7) | 7.2 (1.6) | 8.1 (1.7) | <0.001 |
| Hematoma SRI, median (IQR) | 69.8 (53.7) | 75.9 (57.7) | 56.9 (23.9) | <0.001 |
| Hematoma DCV, median (IQR), % | 12.1 (2.4) | 11.9 (2.1) | 13.5 (2.5) | <0.001 |
| Hematoma location |  |  |  | 0.404 |
| deep hematoma | 273 (75.0) | 216 (73.5) | 57 (81.4) |  |
| lobar hematoma | 70 (19.2) | 59 (20.1) | 11 (15.7) |  |
| cerebellar hematoma | 14 (3.9) | 12 (4.1) | 2 (2.9) |  |
| brainstem hematoma | 7 (1.9) | 7 (2.4) | 0 (0.0) |  |
| IVH | 85 (23.4) | 72 (24.5) | 13 (18.6) | 0.293 |
| CTA spot sign | 29 (8.0) | 15 (5.1) | 14 (20.0) | <0.001 |
| Hypodensities | 169 (46.4) | 125 (42.5) | 44 (62.9) | 0.002 |
| Blend sign | 52 (14.3) | 37 (12.6) | 15 (21.4) | 0.057 |
| Blackhole sign | 43 (11.9) | 30 (10.2) | 13 (18.6) | 0.053 |
| Island sign | 49 (13.5) | 39 (13.3) | 10 (14.3) | 0.822 |
| Satellite sign | 85 (23.4) | 62 (21.1) | 23 (32.9) | 0.036 |
| Heterogeneous | 51 (14.0) | 33 (11.2) | 18 (25.7) | 0.002 |
| Irregular shape | 80 (22.0) | 57 (19.4) | 23 (32.9) | 0.014 |
| Na, median (IQR), mmol/L | 138.2 (3.6) | 138.2 (3.4) | 138.7 (4.0) | 0.774 |
| K, median (IQR), mmol/L | 4.1 (0.7) | 4.1 (0.6) | 4.2 (0.9) | 0.025 |
| Glucose, median (IQR), mmol/L | 7.5 (2.8) | 7.5 (2.6) | 7.4 (3.6) | 0.509 |
| Creatinine, median (IQR), mg/dL | 67.2 (22.7) | 65.3 (21.2) | 71.2 (23.4) | 0.040 |
| ALT, median (IQR), U/L | 20.0 (16.5) | 19.9 (15.5) | 20.5 (16.0) | 0.409 |
| AST, median (IQR), U/L | 21.2 (12.7) | 21.1 (10.0) | 22.7 (17.2) | 0.078 |
| TP, median (IQR), mg/L | 72.3 (7.1) | 72.2 (7.2) | 72.5 (7.7) | 0.128 |
| D-dimer, median (IQR), mg/L | 0.5 (0.3) | 0.5 (0.4) | 0.5 (0.3) | 0.218 |
| BNP, median (IQR), ng/L | 36.5 (63.7) | 36.5 (62.7) | 36.5 (69.1) | 0.623 |
| Leukocyte, median (IQR), 10^9^/L | 9.8 (4.1) | 10.0 (4.0) | 8.3 (3.9) | 0.004 |
| Erythrocyte, median (IQR), 10^9^/L | 4.7 (0.8) | 4.7 (0.8) | 4.7 (0.7) | 0.186 |
| Platelet, median (IQR), 10^9^/L | 217 (68) | 221 (66) | 205 (74) | 0.240 |
| Hemoglobin, median (IQR), g/L | 146 (21) | 146 (23) | 149 (22) | 0.241 |
| Neutrophil, median (IQR), 10^9^/L | 8.1 (3.9) | 7.9 (4.2) | 8.7 (4.8) | 0.075 |
| Lymphocyte, median (IQR), 10^9^/L | 1.3 (0.7) | 1.3 (0.7) | 1.1 (0.7) | 0.008 |
| NLR, median (IQR) | 6.2 (6.0) | 6.2 (6.0) | 8.1 (8.5) | 0.004 |
| CRP, median (IQR), mg/L | 4.1 (8.3) | 3.7 (6.1) | 8.5 (11.1) | <0.001 |

Continuous variables are expressed as the mean ± SD, standard deviation or median (interquartile range, IQR), while categorical variables are expressed as n (%). ALT, Alanine transaminase; AST, Aspartate transaminase; BNP, Brain natriuretic peptide; CHD, coronary heart disease; CT, computed tomography; CTA, computed tomography angiography; GCS, Glasgow Coma Scale; HE, hematoma expansion; HU, Hounsfield Unit; IS, ischemic stroke; IVH, intraventricular hemorrhage, National Institutes of Health Stroke Scale; SBP, systolic blood pressure; SRI, surface regularity index; DCV, density coefficient of variation; BNP, brain natriuretic peptide; TP, total protein.

Table S2. The coefficients in Logistic regression of variables in models.

| Variable | Model 1 | Model 2 |
| --- | --- | --- |
| Intercept | -5.64778629853152 | -8.54423063854948 |
| Leukocyte | -0.172011412489795 | -0.161512850571248 |
| Total protein | 0.0685671042147257 | 0.0572054286126005 |
| NLR | - |  |
| Hemoglobin | - |  |
| Onset to baseline CT | - |  |
| CTA spot sign | 1.51419408903625 | - |
| Hypodensities | 0.7508166220113 | - |
| Heterogeneous | 0.533825367529205 | - |
| Irregular shape | 0.512193794648075 | - |
| Blend sign | 0.557860607090391 | - |
| SRI | - | -0.0204251369395843 |
| DCV | - | 0.475021303123046 |

Note: SRI, surface regularity index; DCV, density coefficient of variation; NLR, neutrophil-lymphocyte ratio; CT, computed tomography, CTA, computed tomography angiography.


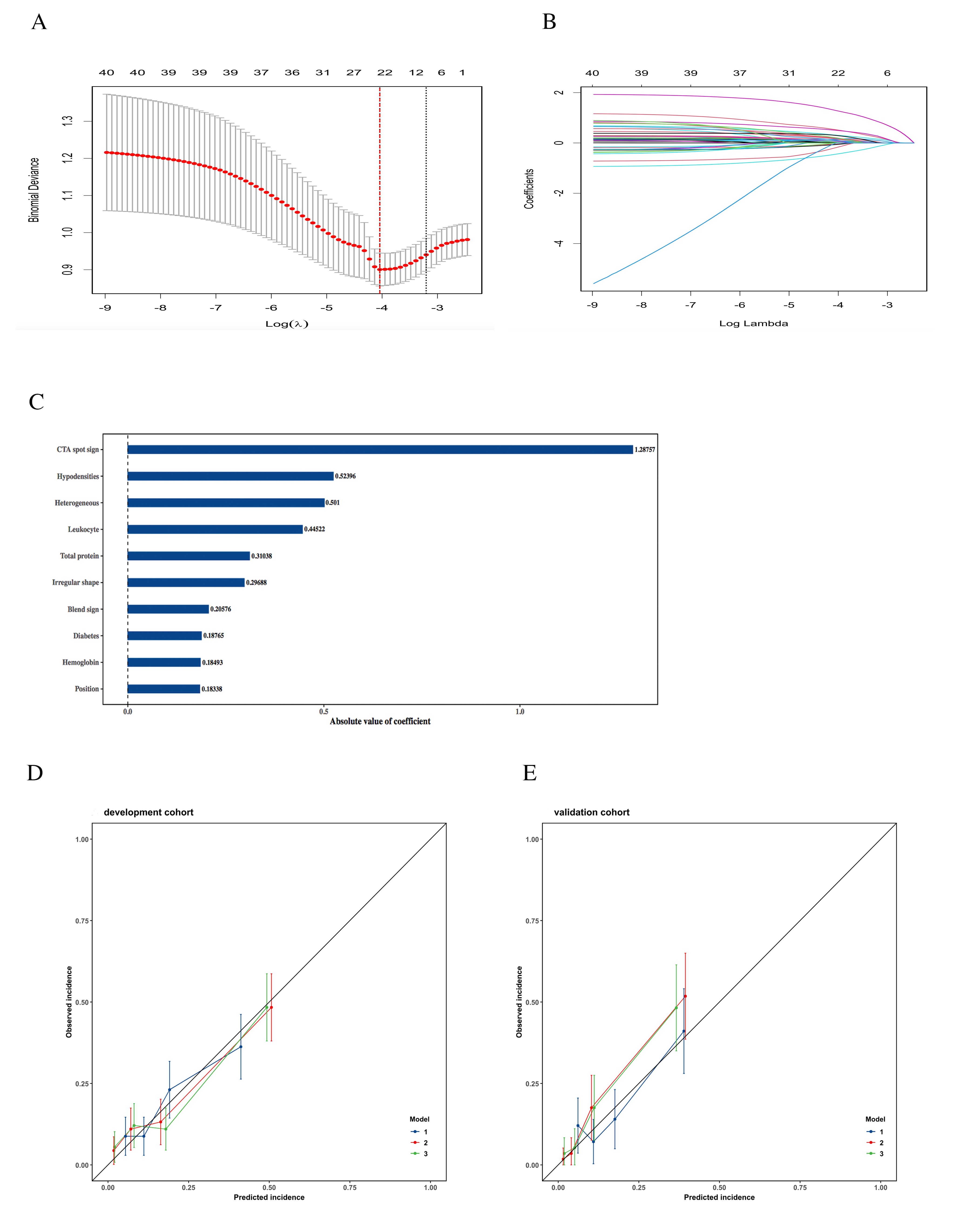
Supplementary Figure S1. Model performance of predictive models.

A. Selection of potential factors to predict HE (model 1); B. Optimal λ based on the deviance was used to select variables with nonzero coefficients (model 1). C. The importance of selected variables was sequenced according to its absolute value of coefficients. Calibration curves of three models for predicting HE in development cohort (D) and validation cohort (E).

CTA, computed tomography angiography; HE, hematoma expansion.


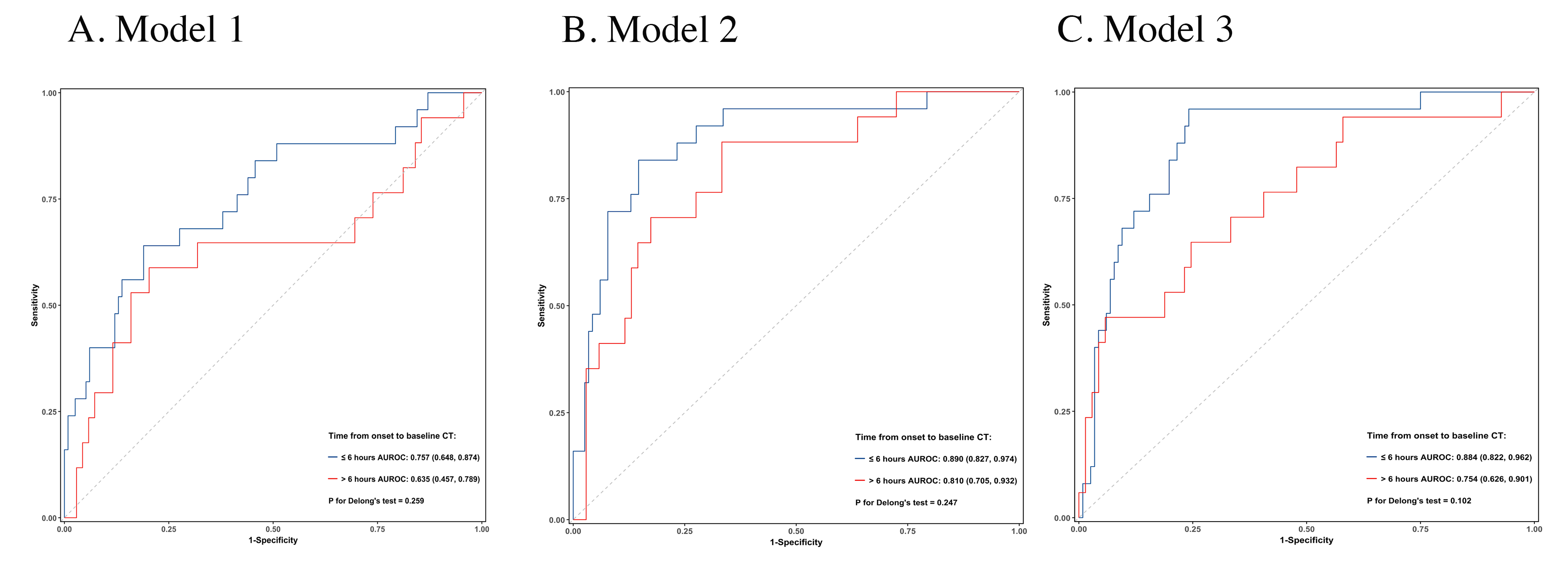


Supplementary Figure S2. The model performances of the subgroup analysis of three predictive models based on whether the time from onset to NCCT is less than 6 hours.

A~C showed the subgroup analysis for Model 1, 2, and 3 of 2 groups (Onset to CT within 6 hours and Onset to CT after 6 hours), and the results showed no statistical difference between the two groups in three models (all the p value >0.05, Delong test).

NCCT, non-contrast computed tomography
